# Efficacy of a novel two-day EMT refresher training program focused on essential EMS knowledge and skills

**DOI:** 10.1186/1865-1380-7-S1-P5

**Published:** 2014-07-25

**Authors:** Pete Acker, Danielle Mianzo, Elizabeth Pirrotta, Matthew Strehlow, Swaminatha Mahadevan

**Affiliations:** 1 Stanford University School of Medicine, Stanford, California, USA

## Objective

A recent study demonstrated surprisingly poor retention of core knowledge and skills in practicing emergency medical technicians (EMTs) in India. The goal of this study was to determine the effectiveness of a novel two-day EMT refresher training course focused on knowledge and skills essential to the proper delivery of prehospital emergency care.

## Methods

Study participants were GVK EMRI EMT instructors who attended the inaugural EMT refresher training course. These participants were from nine states within India and represented mixed educational backgrounds, including doctors and EMTs. After receiving a confidential unique ID, each study participant took a 30 multiple-choice question (MCQ) pre-test and a 60 MCQ post-test before and after the course, respectively. Differences between pre- and post-test scores were analyzed using the Wilcoxon signed rank sum test paired by participant.

## Results

Fifty course participants took both tests. For the pre-test, the mean score was 72.3% (SD 10.6%) and for the post-test, the mean score was 85.3% (SD 9.6%), corresponding to a mean improvement of 13.6% (SD Δ8.3%, p<0.001). Of the thirty topic areas assessed, participants showed the greatest initial competency in cardiac monitoring, CPR, and ventilation (98%, 96% and 92% pre-test averages, respectively). The greatest improvement was seen in extremity hemorrhage control, OPA/NPA usage, and EMS roles and responsibilities (Δ +74%, +42%, +32%, respectively), while decreased performance occurred in documentation, airway obstruction, and ventilation (Δ -18%, -9%, -5%, respectively).

## Limitations

A key limitation of the study was that the MCQs were not validated prior to testing. Furthermore, no control group was available against which to compare the performance of participants.

## Conclusion

This novel two-day refresher training program was an effective teaching tool for EMT instructors leading to improvement in knowledge across numerous key prehospital care content areas. Next steps include assessment of the training course utilizing practicing Indian EMTs.

